# Biodegradability of Poly(hydroxyalkanoate) Materials

**DOI:** 10.3390/ma2031104

**Published:** 2009-08-28

**Authors:** Keiji Numata, Hideki Abe, Tadahisa Iwata

**Affiliations:** 1 Department of Biomedical Engineering, Tufts University / 4 Colby Street, Medford, MA 02155, USA; E-Mail: keiji.numata@tufts.edu (K.N.); 2 Chemical Analysis Team, RIKEN Advanced Science Institute/ Hirosawa 2-1, Wako-shi, Saitama 351-0198, Japan; E-Mail: habe@riken.jp (H.A.); 3 Department of Biomaterial Sciences, Graduate School of Agricultural and Life Sciences, The University of Tokyo / 1-1-1 Yayoi, Bunkyo-ku, Tokyo 113-8657, Japan

**Keywords:** poly(hydroxyalkanoate) (PHA), biodegradation, PHA depolymerase

## Abstract

Poly(hydroxyalkanoate) (PHA), which is produced from renewable carbon resources by many microorganisms, is an environmentally compatible polymeric material and can be processed into films and fibers. Biodegradation of PHA material occurs due to the action of extracellular PHA depolymerase secreted from microorganisms in various natural environments. A key step in determining the overall enzymatic or environmental degradation rate of PHA material is the degradation of PHA lamellar crystals in materials; hence, the degradation mechanism of PHA lamellar crystals has been studied in detail over the last two decades. In this review, the relationship between crystal structure and enzymatic degradation behavior, in particular degradation rates, of films and fibers for PHA is described.

## 1. General Introduction

**Figure 1:**
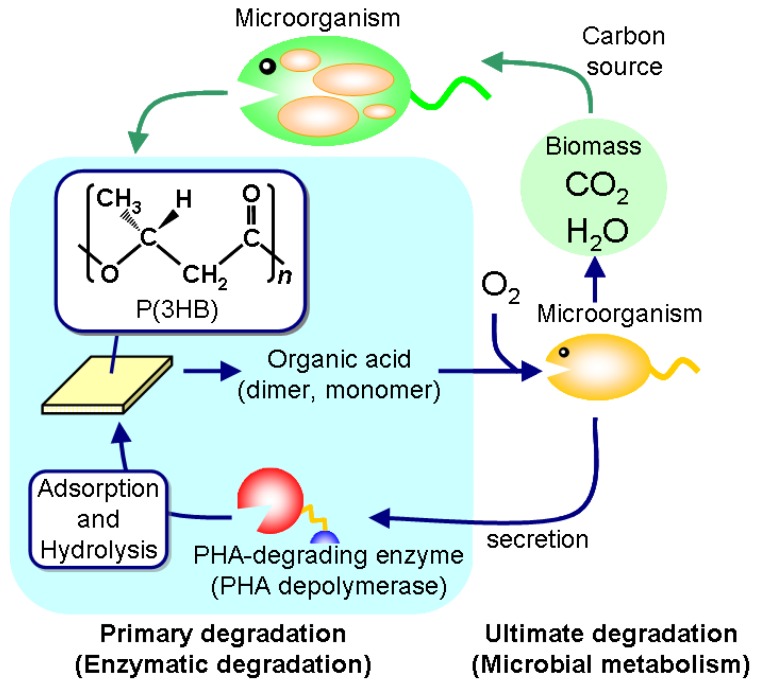
Biosynthesis and biodegradation process of PHA in a natural environment.

Polyesters are now universally used as fibers and films in various areas, while plastic-waste management recently became a critical problem of global environment. Bio-based and biodegradable polyesters have therefore been in demand in order to reduce carbon dioxide emissions from plastic waste as well as to build a sustainable society. Poly(hydroxyalkanoate)s (PHAs), one such eco-friendly polymeric materials, are polyesters synthesized by a variety of bacteria as an intracellular storage material of carbon and energy (Figure 1) [1,2,3,4,5]. Poly[(*R*)-3-hydroxybutyrate] [P(3HB)] was first isolated from *Bacillus megaterium* in the 1920s and later identified as a microbial reserve polyester [6]. P(3HB) was initially recognized as a material that was brittle and stiff material over a long period, and therefore it not interchangeable with polyethylene (PE) or polystyrene (PS). The tensile strength, elongation to break, and Young’s modulus of P(3HB) fibers were reported to be 0.19 GPa, 54%, and 5.6 GPa [7]. The identification of hydroxyalkanoate (HA) units other than 3HB unit as constituents of microbial reserve polyesters proved to have a major impact on research and commercial interests in this microbial polyester since the incorporation of different HA units into P(3HB) can vary its mechanical and thermal properties [8,9,10]. Additionally, Iwata and coworkers recently succeeded in improving its mechanical properties by using new drawing techniques, and the resulting tensile strength, elongation to break, and Young’s modulus of the drawn PHA fiber were 1.32 GPa, 35%, and 18.1 GPa [11]. These mechanical properties of PHA are comparable to PE and polypropylene (PP) of industrial level as well as poly(glycolic acid) which is used for sutures. Thus, PHA has become a much more attractive material with respect to mechanical strength. 

One of the unique properties of PHA materials is their biodegradability in natural environments [12,13]. PHA is a high-molecular-weight solid polymer that cannot be transported through the cell wall, so microorganisms such as bacteria and fungi excrete extracellular PHA-degrading enzymes (PHA depolymerases) that hydrolyze the solid PHA into the water-soluble monomer and oligomers. These low-molecular-weight degradation products are then transported into the cell and subsequently metabolized as carbon and energy sources.

The structures and properties of several of these PHA depolymerases have had characterized by many groups [14,15,16,17,18,19,20,21,22,23,24,25,26,27,28,29]. The PHA-degrading microorganisms and enzymes were first isolated from *Pseudomonas* strains by Chowdhury in 1963 [14]. The microorganisms excrete a number of extracellular PHA depolymerases to degrade environmental PHA and utilize the decomposed compounds as nutrients. Several PHA depolymerases have also been purified from some microorganisms such as *Pseudomonas lemoignei* [19], *P*. *stutzeri* [20], *Ralstonia pickettii* T1 (formally known as *Alcaligenes faecalis*) [21,22], *Comamonas testosteroni* [23], and *C*. *acidovorans* [24]. Analyses of the structural genes of the extracellular PHA depolymerase from *R*. *pickettii* T1, which is the most studied PHA depolymerase, have shown them to have a multi-functional domain structure, *i*.*e*., a catalytic domain, a substrate-binding domain, and a linker region, connecting the two domains [22,25,26]. The substrate-binding domain is primarily responsible for the adsorption of the PHA depolymerase to the surface of water-insoluble polyester materials. In recent years, it has also been proposed that the substrate-binding domain is of great importance in the degradation process disturbing molecular chain packing at the surface of P(3HB) crystals, which induces the initial step of the enzymatic degradation [29,30,31,32,33,34,35,36,37,38]. The binding interactions of the single substrate-binding domain of PHA depolymerase from *R*. *pickettii* T1 on P(3HB) thin film were estimated to be about 100 pN by atomic force microscopy (AFM) force-curve measurements [39]. The substrate specificity of PHA depolymerase was first investigated using various hydrolases and polymer substrates [40], demonstrating that PHA depolymerases show relatively narrow substrate specificity for the hydrolysis reactions of PHAs. It is also suggested that the active site of PHA depolymerase from *R*. *pickettii* T1 recognizes at least three monomeric units as a substrate [40,41,42,43]. Substrate-binding specificity, in addition to substrate-hydrolysis, was also studied using modified PHA depolymerase and various substrates by means of AFM and quartz crystal microbalance (QCM) by Doi and coworkers [38,41,44,45,46]. Based on the hydrophobic characteristics and density of the ester bonds at the surface of polyester crystals, the amount of adsorbed enzyme may be dependent on the number of ester bonds rather than the hydrophobicity of the surface. Equally, the PHA depolymerase binds to aliphatic polyesters not only by hydrophobic interaction but also by specific interactions between the ester bonds of polyesters and the binding domain.

**Figure 2:**
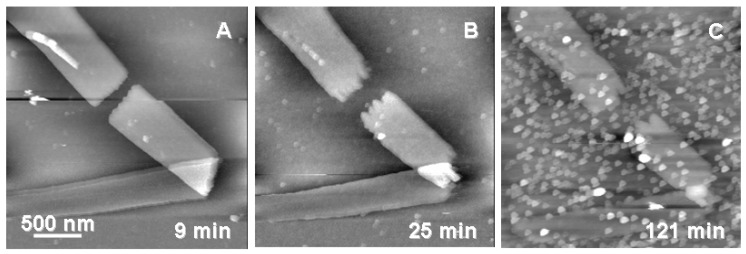
Adsorption and initial degradation of P(3HB) single crystals by PHA depolymerase from *R*. *pickettii* T1 [51]. Reproduced with permission from Numata *et al*., *Biomacromolecules*; published by ACS, 2007.

Rates of enzymatic degradation of P(3HB) by PHA depolymerase are strongly dependent on the crystalline state of PHA, as well as the concentration, properties, and reaction conditions of PHA depolymerase [47,48,49]. The rate of enzymatic hydrolysis increased to a maximum value with the concentration of PHA depolymerase, followed by a gradual decrease. Based on the kinetic analyses of the enzyme adsorption on the surface of P(3HB), it has been confirmed that the adsorption isotherms of PHA depolymerase are expressed by the Langmuir adsorption equation [50]. Recently, Yamashita *et al*. suggested that each molecule of PHA depolymerase adsorbs irreversibly on the surface of the P(3HB) film, and is then easily substituted by the attack of the enzyme molecules in the solution [46]. The apparent adsorption isotherms of PHA depolymerase therefore seems to obey the Langmuir isotherm. Also, it was confirmed the irreversible adsorption manner of PHA depolymerase from *R*. *pickettii* T1 on the P(3HB) crystals using the technique to visualize enzyme molecules by AFM as shown in Figure 2 [51]. 

The degradation of the amorphous region is much faster compared with the crystalline region of PHA, therefore a key step to determine the degradation rate of the overall material is the degradation of the crystalline region. The enzymatic degradation rate is also influenced by the crystalline state, such as crystallinity, crystal size, and lamellar thickness of PHA materials. The mechanism of degradation of PHA lamellar crystals has therefore been investigated by many groups [22,42,48,51,52,53,54,55,56,57]. In this review, we have attempted to give an overview of the degradation mechanism of PHA lamellar crystals as well as the relation between crystal structure and enzymatic degradation behavior, especially degradation rates, of films and fibers for PHA.

## 2. Enzymatic Degradation Process of PHA Lamellar Crystals

**Figure 3:**
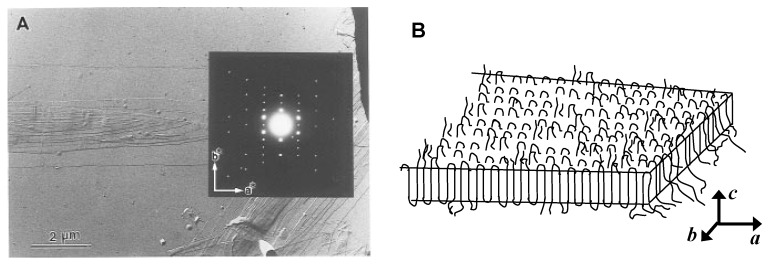
Morphology and crystalline structure of P(3HB) single crystals. (A) Electron micrographs of P(3HB) single crystals. (B) Schematic model of molecular chain packing of P(3HB) single crystals [42]. Reproduced with permission from Iwata *et al*., *Macromolecules*; published by ACS, 1997.

The degradation rate of PHA materials is strongly dependent on their solid state properties such as crystallinity, lamellar thickness, and crystal sizes, which are affected by chemical structure. Many researchers therefore have investigated the degradation mechanism of PHA lamellar crystals using PHA single crystals [22,42,48,51,52,53,54,55,56,57]. Single crystals, which clearly have a uniform and well-defined structure, are an excellent monolamellar system for studying the enzymatic degradation process. PHA single crystals have been prepared from many kinds of solvents, and their crystalline morphology and structure were investigated through the use of microscopic analyses [42,48,52,53,54,55,56,57]. Typically, P(3HB) forms a lath-shaped crystal with dimensions of around 0.3–2 µm and 5–10 µm along the short and long axes, respectively (Figure 3A). Based on the electron diffractogram of P(3HB) single crystals, the long axis is the crystallographic *a* axis (Figure 3B). The thickness of P(3HB) single crystals ranges from 4–10 nm depending on the molecular weight, solvent, and crystallization temperature.

**Figure 4:**
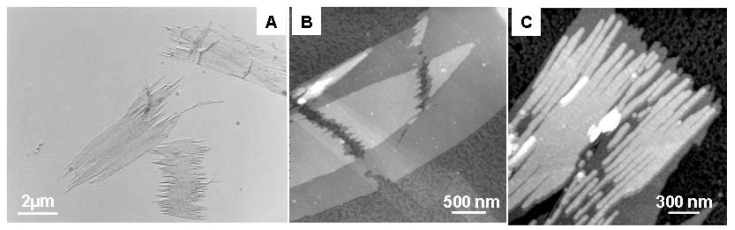
P(3HB) single crystals after enzymatic degradation by the PHA depolymerase from *R*. *pickettii* T1 at 37 ºC [42,55]. (A) Electron micrograph of P(3HB) single crystals after 80 min of the enzymatic degradation. (B,C) AFM height images of P(3HB) single crystals after 60 (B) and 90 min (C) of the enzymatic degradation. Reproduced with permission from Iwata et al. and Murase *et al*., *Macromolecules*; published by ACS, 1997 and 2001.

PHA depolymerases from bacteria and fungi have been used to study the enzymatic degradation of P(3HB) single crystals in order to elucidate the degradation mechanism of the crystalline region for P(3HB). Marchessault and co-workers were the first to perform the enzymatic degradation of P(3HB) single crystals by PHA depolymerases [52,53,54]. They evaluated the degradation behavior by turbidimetric and titrimetric assays as well as by monitoring the changes in molecular weight of the polymer [52]. No decrease in molecular weight was observed in the partly degraded polymer, suggesting preferential degradation from the crystal edges rather than the chain folds of the lamellar surface and supporting the hypothesis of a combined *endo* and *exo* degradation mechanism by the fungus *Aspergillus fumigatus* and the bacterium *Pseudomonas lemoignei* [52]. Nobes *et al.* used transmission electron microscopy (TEM) to show the conversion of PHA single crystal into needlelike morphologies after enzymatic degradation [53]. Iwata and co-workers investigated the enzymatic degradation of single crystals of P(3HB) and its copolymer single crystals by TEM and AFM (Figure 4) [42,48,55,56]. All these researchers reported that the single crystals were enzymatically hydrolyzed preferentially at the crystal edges (*ac* plane) and ends (*bc* plane) rather than at the chain-folding surfaces (*ab* plane) of the single crystals. 

**Figure 5:**
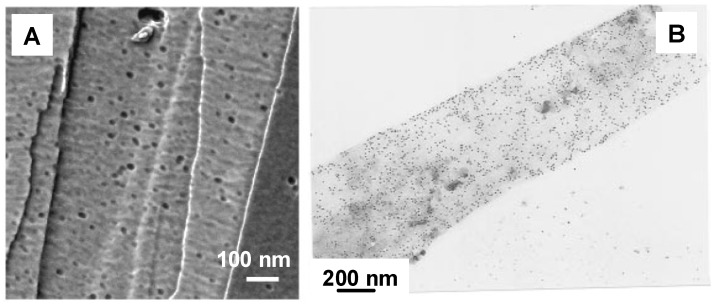
PHA depolymerases from *R*. *pickettii* T1 adsorbed on the surface of P(3HB) single crystals [37,42]. (A) AFM phase image of the PHA depolymerase on P(3HB) single crystal in air. (B) Visualization of the adsorption on P(3HB) single crystal by gold labeling and transmission electron microscopy. Reproduced with permission from Numata *et al*. and Iwata *et al*., *Macromol. Biosci*. and *Macromolecules*; published by Wiley-VCH Verlag GmbH & Co. KGaA and ACS, 2006 and 1997.

The binding specificity of PHA depolymerase to PHA single crystals has also been evaluated by using immuno-gold labeling techniques or AFM observation (Figure 5) [36,37,38,42,48,51]. Black dots in Figure 5A and Figure 5B denote single PHA depolymerase molecules. These results demonstrate that PHA depolymerase adsorbs randomly onto the surface of P(3HB) single crystals without any preference towards crystalline edges, ends, or surfaces. 

The lamellar thickness and molecular weight of P(3HB) crystals remained the same during the enzymatic degradations. The enzymatic reaction produced many narrow cracks and small crystal fragments along the crystal long axis corresponding to the crystallographic *a* axis of P(3HB) single crystals independent of both surface morphology of the single crystals and the types of PHA depolymerases. This is because loose-chain packing regions, which exist along the *a* axis with higher molecular mobility, preferentially degrade rather than the tight-chain packing region by PHA depolymerases [57,58,59]. As for the single crystals of PHA copolymers, the enzymatic degradation mechanism was found to be the same as in the case of P(3HB) single crystal.

**Figure 6:**
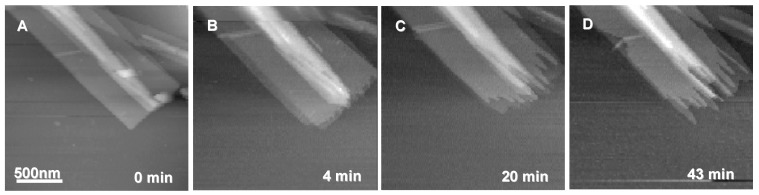
Real-time AFM height images of P(3HB) single crystals before (A) and during enzymatic degradation over time by the PHA depolymerase from *R*. *pickettii* T1 at 37 ºC [57]. Reproduced with permission from Numata *et al*., *Biomacromolecules*; published by ACS, 2005.

**Figure 7:**
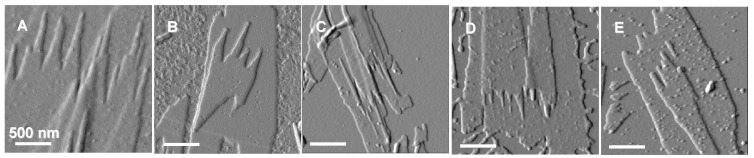
AFM amplitude images of PHA single crystals for (A) high-molecular-weight P(3HB) (*M*n 240000), (B) middle-molecular-weight P(3HB) (*M*n 55000), (C) low-molecular-weight P(3HB) (*M*n 8500), (D) P(3HB-*co*-6mol%-3HV), and (E) P(3HB-*co*-14mol%-3HV) after enzymatic degradation by PHA depolymerase from *R*. *pickettii* T1 for 1h at 37 ºC [57]. Reproduced with permission from Numata *et al*., *Biomacromolecules*; published by ACS, 2005.

We recently performed AFM studies on a quantitative characterization of the dimensions of the fibril-like crystals generated at the edge of lamellar crystals during enzymatic degradation using PHA depolymerase secreted from *R*. *pickettii* T1 [57,61]. The real-time AFM imaging of the lamellar crystals during enzymatic degradation has been investigated [57,61]. Figure 6 shows typical real-time AFM height images of P(3HB) single crystals during enzymatic degradation. Enzymatic erosions proceeded from the edges and ends of lamellar crystals, and the fibril-like crystals were obviously generated gradually by the action of PHA depolymerase. Figure 7 shows AFM amplitude images of 5 types of PHA single crystals after degradation for 1 h. The copolymers used in this study were poly[(*R*)-3-hydroxybutyrate-*co*-6mol%-(*R*)-3-hydroxyvalerate] (P(3HB-*co*-6mol%-3HV)) and poly[(*R*)-3-hydroxybutyrate-*co*-14mol%-(*R*)-3-hydroxyvalerate] (P(3HB-*co*-14mol%-3HV)). The AFM observations of single crystals for 5 types of PHAs after the degradation were performed in order to clarify differences in morphologies of fibril-like crystals among different second monomer compositions and molecular weights. Each of the image in Figure 7 shows that the fibril-like crystals were formed along the long axis of all single crystals after enzymatic degradation. The results also revealed the effect of both molecular weight and second-monomer composition on the morphology of fibril-like crystals. 

The thickness of the major part of the remaining crystals was almost identical with the crystals before degradation, as reported previously [42,53]. Based on our studies, the thickness at the root of fibril-like crystals was also almost identical to the thickness of single crystals before degradation. However, we found that the thickness at the tip of the fibril-like crystals was apparently thinner than the thickness of the single crystals before degradation. It is of interest to note that the fibril-like crystals did not taper completely whereby, we did not observe any crystals with dimensions of either less than 1.9 nm thickness or less than 28 nm width for all PHA samples after degradation at 37 ºC for 1 h. These minimum values of thickness and width of fibril-like crystals may represent the critical dimensions to maintain the crystalline lamellar structure of P(3HB) by the intermolecular-chain-packing. 

As shown in Figure 7, the grooves were formed along the crystallographic *a* axis, and the erosions of fibril-like crystals progressed from both the tip of crystals along the *a* axis and the edges along the *b* axis. The eroded distances at the tip and edge of fibril-like crystals and at the point of grooves were determined from the real-time AFM images by measuring distances from the fixed spot in the images to the edges and ends of eroding crystals. Table 1 lists the erosion rates at the tip and edge of fibril-like crystals and the point of grooves and overall erosion rates of PHA single crystals. For the single crystals of P(3HB) with molecular weight of 8,500, the grooves grew along the crystallographic *a* axis at a rate of 10.6 nm/min. The fibril-like crystals were eroded from the tip of crystals along the *a* axis at a rate of 3.2 nm/min and from the edges along the *b* axis at a rate of 1.1 nm/min. 

**Table 1:** Partial and overall erosion rates along the *a* and *b* axes at the tip and root of the fibril-like crystals during enzymatic degradation [57]. Reproduced with permission from Numata *et al*., *Biomacromolecules*; published by ACS, 2005.

Sample	Partial erosion rates *^a^*, Nm/min			Overall erosion rates, mg/(min·cm^2^)	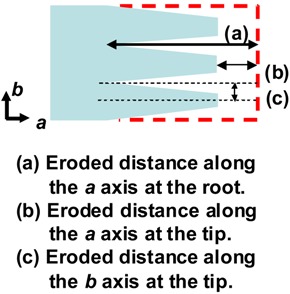
	Root	Tip			
	*a* axis	*a* axis	*b* axis		
*^b^* H-P(3HB)	3.7	2.6	1.3	0.0007	
M-P(3HB)	8.3	3.8	1.3	0.0015	
L-P(3HB)	10.6	3.2	1.1	0.0018	
P(3HB-*co*-6mol%-3HV)	12.4	6.8	2.0	0.0026	
P(3HB-*co*-14mol%-3HV)	19.7	14.1	2.1	0.0065	

*^a^* Erosion rates were determined from real-time AFM observation as shown in Figure 6. *^b^* H-P(3HB), M-P(3HB) and L-P(3HB) mean high-molecular-weight (*M_n_*:240,000), middle-molecular-weight (*M_n_*:55,000) and low-molecular-weight P(3HB) (*M_n_*:8,500), respectively.

For the single crystals of P(3HB) with different molecular weights, the erosion rate at the point of the grooves along the *a* axis increased with a decrease in the molecular weight of P(3HB), while those at the tip of fibril-like crystals along the *a* and *b* axes revealed relatively similar values, independent of molecular weight. As mentioned above, the formation of grooves is attributed to the degradation of disordered chain-packing region in single crystals. The number of the chain ends increases on the crystalline surface with a decrease in molecular weight of P(3HB). With an increase in chain end groups, the disordered chain-packing region may become more unstable. Additionally, PHA depolymerase easily hydrolyzes the P(3HB) molecules from the chain ends exposed on the surface of the single crystal. The erosion rate at the grooves (disordered chain-packing region) therefore increased with a decrease in the molecular weight of P(3HB). In contrast, the erosion rates along the *a* and *b* axes at the tip of fibril-like crystals was similar, independent of the molecular weight, for all P(3HB) samples. All the fibril-like crystals consist of highly ordered chain-packing region. Further, the erosion rate along the *a* axis of fibril-like crystals reflected the degradation rate at the tip of the crystals. The packing structure of such highly ordered crystalline regions may be slightly influenced by the numbers of chain-foldings and chain ends. For poly[(*R*)-3-hydroxybutyrate-*co*-(*R*)-3-hydroxyvalerate] [P(3HB-*co*-3HV)] samples, both the erosion rates at the point of the grooves and at the tip of fibril-like crystals along the *a* axis increased significantly with an increase in the 3HV composition, whereas the rates at the edges along the *b* axis were slightly increased by the introduction of 3HV units. 

The enzymatic erosion rate of PHA single crystals was also estimated by the volumetric analysis from real-time AFM height images [57]. By considering the density of the P(3HB) crystalline region, the changes in the volume of single crystals during enzymatic degradation can be converted into changes in weight. Table 1 shows the rate of erosion for PHA single crystals determined by weight during enzymatic degradation. The results of overall erosion rates from AFM measurements were comparable to the enzymatic erosion rates of melt-crystallized films reported by Abe *et al*. [41], implying that we succeeded in analyzing the overall erosion rate of PHA single crystal during enzymatic degradation by real-time AFM observation. For P(3HB) single crystals, the overall erosion rates increased with a decrease in the molecular weight of P(3HB), similar to the erosion rate at the grooves. The overall degradation rates of P(3HB-*co*-3HV) single crystals were much faster than those of P(3HB) single crystals. Thus, we succeeded in estimating the enzymatic erosion rate of the crystalline region from real-time AFM height images.

**Figure 8:**
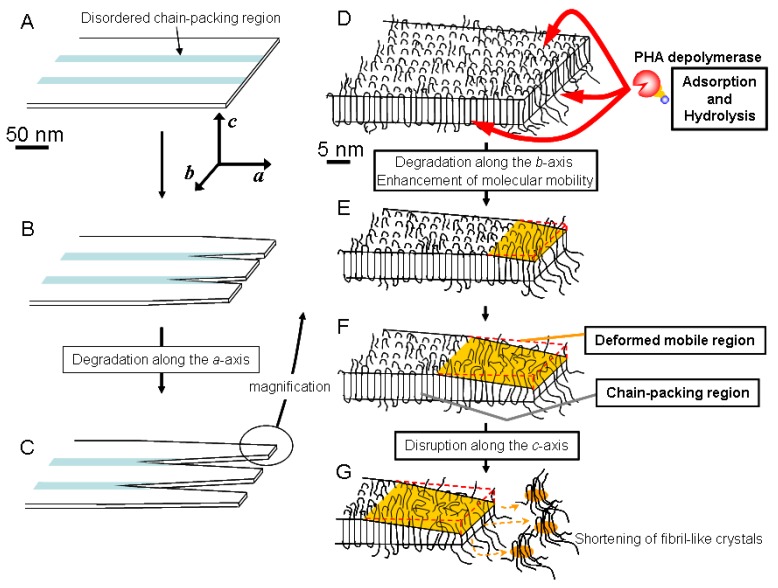
Schematic model of the enzymatic degradation process of PHA lamellar crystals by PHA depolymerase from *R*. *pickettii* T1 [58]. Reproduced with permission from Numata *et al*., *Biomacromolecules*; published by ACS, 2005.

Based on these results, we have proposed a mechanism for the enzymatic degradation mechanism of fibril-like crystals generated from PHA single crystals, as shown in Figure 8 [57]. The PHA single crystals may consist of both ordered and disordered chain-packing regions that exist in periodic manner due to the crystallization characteristic of P(3HB) molecules (Figure 8A) [58,59,60]. PHA depolymerases adsorb homogeneously on the edges, ends and chain-folding crystalline surfaces. The adsorbed enzymes predominantly hydrolyze the polymer molecules at the disordered chain-packing regions in single crystals from the edges and ends, as shown in Figure 8B. Then, the ordered chain-packing regions remain as fibril-like crystals (Figure 8C). Figure 8E demonstrates that the enzymes adsorbed on the fibril-like crystals also erode the ordered chain-packing region from the edge, which results in the tapering of the fibril-like crystals down to a minimum width of approximately 28 nm. In addition, the adsorption of enzyme on the fibril-like crystals also may enhance the molecular mobility in ordered chain-packing regions. Subsequently, the molecular packing structure in the ordered chain-packing region is gradually distorted starting from the chain-folding surface along the crystallographic *c* axis to reach a minimum value (around 1.9 nm), as shown in Figure 8F. Tapping mode AFM was used for the dimensional analysis of PHA single crystals. The remaining ordered chain-packing region will be progressively eroded from the edge by the catalytic action of the enzyme. At the same time, adsorption of the enzyme on the chain-folding region will produce surface distortion. Together, both these effects will reduce the crystal dimensions to the minimum values at which, the lamellar structure at the tip of the fibril-like crystal will collapse similar to the tip of a glacier (Figure 8G). As a result of these degradation processes, the tip of the fibril-like crystals will fragment into smaller pieces, which will eventually be hydrolyzed to water-soluble dimers and monomers by the PHA depolymerase.

## 3. Biodegradability of PHA Films

PHA films with a thickness of several hundred micrometers are composed of lamellar crystalline and amorphous regions. Melt-crystallized and/or drawn PHA films show higher mechanical properties, higher crystallinity, and larger lamellar crystals in comparison with solvent-cast PHA film. Here, we review relations between the degradation rate of various PHA films and the crystalline and chemical structures of PHA. 

**Figure 9:**
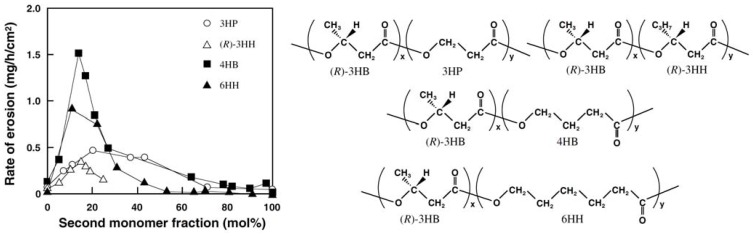
Effects of second monomer units on the rate of enzymatic erosion of solution-cast films of PHA by PHA depolymerase from *R*. *pickettii* T1 [43]. Reproduced with permission from Abe and Doi, *Int. J. Biol. Macromol.*; published by Elsevier, 1999.

The effects of the chemical structure of second monomer units and copolymer compositions on the rate of enzymatic erosion were investigated through the enzymatic degradation of solution-cast films of PHA with various hydroxyalkanoate units in the presence of PHA depolymerase. The enzymatic degradations of the solution-cast films of P(3HB) homopolymer [62] and five types of copolyesters; poly[(*R*)-3-hydroxybutyrate-*co*-(*R*)-3-hydroxyvalerate] (P(3HB-*co*-3HV)) [63], poly[(*R*)-3-hydroxy-butyrate-*co*-3-hydroxypropionate] (P(3HB-*co*-3HP)) [64], poly[(*R*)-3-hydroxybutyrate-*co*-(*R*)-3-hydroxyhexanoate] (P(3HB-*co*-3HH)) [63], and poly[(*R*)-3-hydroxybutyrate-*co*-4-hydroxybutyrate] (P(3HB-*co*-4HB)) [65], poly[(*R*)-3-hydroxybutyrate-*co*-6-hydroxyhexanoate] (P(3HB-*co*-6HH)) [66], were performed in aqueous solutions of purified PHA depolymerase secreted from *R*. *pickettii* T1 at 37 °C [67]. The enzymatic degradation occurred on the surface of PHA films, resulting weight loss of the films which increased proportionally with time. Figure 9 shows the rates of enzymatic erosion of PHA films by PHA depolymerase from *R*. *pickettii* T1. The rate of enzymatic erosion on the solution-cast PHA films increased markedly with an increase in the fraction of second monomer units up to 10–20 mol% to reach a maximum value followed by a decrease in the erosion rate. The highest rates of enzymatic erosion were 5 to 10 times larger in comparison to P(3HB) homopolymer films. 

**Figure 10:**
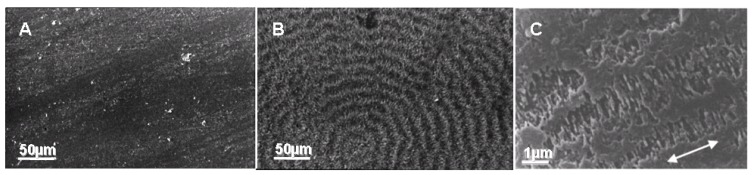
SEM images of the surfaces of the melt-crystallized PHA film before and after enzymatic degradation by PHA depolymerase from *R*. *pickettii* T1. (A) P(3HB-*co*-6HH) film before enzymatic degradation [41]. (B) P(3HB-*co*-6HH) film after enzymatic degradation for 30 min [41]. (C) Stretched-annealed UHMW-P(3HB) film after enzymatic degradation for 3 h. The arrow indicates the draw direction [74]. Reproduced with permission from Abe *et al.* and Kusaka *et al.*, Macromolecules and *Int. J. Biol. Macromol.*; published by ACS and Elsevier, 1998 and 1999.

P(3HB) is a semicrystalline thermoplastic with a melting temperature around 180 °C. The melt-crystallized films of P(3HB) exhibit large banded spherulites of 50–500 mm in diameter, and the spherulitic morphology and degree of crystallinity are dependent upon the crystallization conditions [68,69]. PHA depolymerase from *R*. *pickettii* T1 was reported to hydrolyze predominantly P(3HB) chains in the amorphous state on the surface of film followed by erosion of chains in the crystalline state [70]. In addition, the rate of enzymatic erosion of P(3HB) film increased with a decrease in the crystallinity, while the size of spherulites did not affect the rate of hydrolysis. Figure 10 shows typical scanning electron micrographs (SEMs) of the surfaces of melt-crystallized P(3HB-*co*-6HH) films before (A) and after enzymatic degradation (B). Before enzymatic degradation, the surface of melt-crystallized films was almost flat and smooth. After enzymatic degradation, the surface was apparently blemished with time by the action of PHA depolymerase, and the ringed texture of spherulites was detected. Two different types of planes can be observed on the surface of PHA spherulites after enzymatic degradation, namely the smooth and rough planes exist alternately along the radial direction of spherulites. The band distance of spherulites determined from the SEMs of films after enzymatic degradation increased with an increase in the crystallization temperature, and the values were identical with the values of band spacing determined from the optical micrographs of spherulites. A similar ringed texture of P(3HB) spherulite could be observed on the surface of the melt-crystallized film exposed in activated sludge [71]. The banded morphology of spherulites is known to arise from the difference in orientation of crystalline axis due to the twisting of lamellar crystals. Both the chain-folding plane and crystal edge plane of lamellae therefore appear on the surface of melt-crystallized polyester films. PHA depolymerase may predominantly hydrolyze the polymer chains at the edge of lamellar stacks on the surface of melt-crystallized films. Figure 10C shows an SEM of a stretched ultra-high molecular-weight P(3HB) (UHMW-P(3HB)) film after enzymatic degradation for 3 h, and this micrograph reveals the non-etched core along the drawing direction and the lamellar crystals perpendicular to the core. The surface morphologies are likely to be in the shish-kebab structure as found in polyethylene crystallized in agitated solution, extruded high modulus polyethylene fibers, extruded nylon-6,6 fibers, and crystallized natural rubber under strain.[69,71,72,73] The high tensile strength of the stretched film is due to the stretched chain core having this shishikebab morphology. In the case of P(3HB) copolymers, an almost similar enzymatic degradation phenomena and morphologies can be observed in the rate of erosion profiles and scanning electron micrographs [23]. 

**Figure 11:**
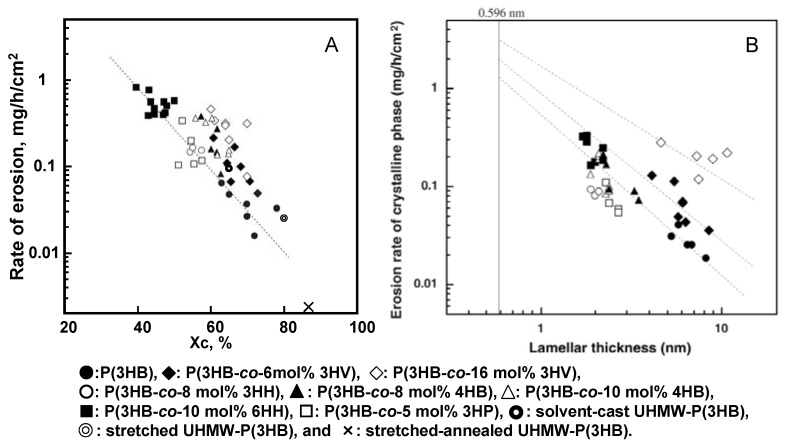
Relation between the rate of enzymatic erosion and the degree of crystallinity (Xc) (A) and the lamellar thickness (B) for melt-crystallized PHA films and solvent-cast, stretched, and stretched-annealed UHMW-P(3HB) films. [41,74] Reproduced with permission from Abe *et al.*, *Macromolecules*; published by ACS, 1998.

Melt-crystallized films of polyesters; P(3HB), P(3HB-*co*-6mol%-3HV), P(3HB-*co*-16mol%-3HV), P(3HB-*co*-8mol%-3HH), P(3HB-*co*-8mol%-4HB), P(3HB-*co*-10mol%-4HB), P(3HB-*co*-10mol%-6HH), and P(3HB-*co*-5mol%-3HP), which were prepared by isothermal crystallization from the melt at different temperatures of 30–140 °C for 3 days, showed well-developed and volume-filled spherulites [41,75]. The degree of crystallinity and lamellar thickness of melt-crystallized PHA films varied from 40 to 78% and from 1.7 to 10.8 nm, respectively, depending both on the chemical structure of second monomer units and on the crystallization temperature [41]. Figure 11A shows the relationship between the rate of enzymatic erosion and degree of crystallinity of melt-crystallized polyester films. The rate of enzymatic erosion of melt-crystallized polyester films significantly decreased as the degree of crystallinity increased. The enzymatic degradation rates of solvent-cast, stretched, and stretched-annealed films of ultra-high molecular-weight P(3HB) (UHMW-P(3HB)) showed the relationship between the rate of enzymatic erosion and the crystallinity for P(3HB) films [74]. In spite of the addition of stretching and annealing procedures, all the rates of erosion were in line as shown in Figure 11A. It is important to note that the rate of erosion is independent of the molecular weight, the second unit compositions, and the processing procedures, but is dependent on the crystallinity. However, it is noted from the result of Figure 11A that the rates of enzymatic erosion for P(3HB-*co*-3HV) films are several times higher than those of P(3HB) films, when the rates are compared with films of an identical crystallinity. The significant difference in the erosion rates for melt-crystallized films of P(3HB) homopolymer and P(3HB-*co*-3HV) copolymers could not be explained only in terms of degree of crystallinity. 

The rates of film erosion of P(3HB-*co*-10mol%-6HH) samples with 40–50% crystallinity [0.396–0.840 mg/(h·cm^2^)] were several orders of magnitude larger than those of P(3HB) samples with 63–78% crystallinity [0.016–0.066 mg/(h·cm^2^)] [41]. This result indicates that the erosion rate of amorphous phase is much larger than that of crystalline phase. Also, we point out that the erosion rate of the crystalline phase is strongly dependent on the lamellar thickness (*l*c) of melt-crystallized copolyester films, as shown in Figure 11B. The erosion rate of the crystalline phase in melt-crystallized films increased markedly with a decrease in the lamellar thickness. The binding of PHA depolymerase on the edge of P(3HB) lamellar crystal may cause an increase in the mobility of polyester chains along the crystal edge, resulting in the formation of disordered P(3HB) chains like polymer chains in an amorphous phase which are easily attacked by the active site of enzyme. The polyester chains exposed on the edge of relatively thin crystalline lamellae may more easily form the disordered polyester chains, rather than those on the edge of relatively thick crystalline lamellae. Accordingly, the erosion rate of crystalline phase may increase with a decrease in lamellar thickness. It is noted from Figure 11B that the erosion rates of crystalline phase in P(3HB-*co*-3HV) films are several times higher than those of P(3HB) films of an identical lamellar thickness and increase with an increase in 3HV fraction. P(3HB-*co*-3HV) copolymers have been known to show isodimorphism due to the cocrystallization of 3HB and 3HV units in the composition range from 0 to 30 mol% of 3HV units [76] and that a portion of 3HV units is included in the P(3HB) crystalline lattice. On the basis of these results, the molecular chains of P(3HB-*co*-3HV) on the edge of crystalline lamellae may be more mobile than the chains of P(3HB) homopolymer on the crystal edge, resulting in more facile erosion by PHA depolymerase being observed on the isodimorphic crystals of P(3HB-*co*-3HV).

Biochemical Oxygen Demand (BOD) biodegradability of PHA films in Arakawa river water (Japan) at 25 ºC was reported by Doi and coworkers as shown in Table 2 [74,77]. The degradation of PHA films was initiated after around 4 days, and then the BOD-biodegradation of P(3HB) increased with time to reach 80% biodegradability in 28 days. The P(3HB-*co*-3HV) copolymers in the composition range of 12-21 mol% 3HV units showed higher BOD-biodegradabilities than P(3HB), and their biodegradabilities reached a constant value, 75 ± 3%, within 14 days. After 28 days, all samples listed in Table 2 except P(3HB-*co*-80mol%-3HV) and P(3HP) completely degraded. Also, the BOD-biodegradability in Arakawa river water demonstrated no influence on the crystallinity and the long period of PHA films [74].

**Table 2:** BOD-biodegradabilities for 28 days and X-ray crystallinities of PHA films [74,77].

Sample	Type of film	Crystallinity,*^a^* %	BOD-Biodegradability, %
P(3HB)	Solvent-cast	60 ± 5	75 ± 8
P(3HB-*co*-4mol%-3HV)		57 ± 5	77
P(3HB-*co*-12mol%-3HV)		50 ± 5	75
P(3HB-*co*-14mol%-3HV)		54 ± 5	76 ± 3
P(3HB-*co*-21mol%-3HV)		59 ± 5	74 ± 2
P(3HB-*co*-80mol%-3HV)		52 ± 5	9 ± 2
P(3HB-*co*-5mol%-4HB)		50 ± 5	80 ± 2
P(3HB-*co*-20mol%-4HB)		38 ± 5	84 ± 4
P(3HB-*co*-56mol%-4HB)		10 ± 5	63 ± 1
P(4HB)		34 ± 5	82 ± 2
P(3HB-*co*-15mol%-3HP)		40 ± 5	70 ± 2
P(3HB-*co*-36mol%-3HP)		19 ± 5	85 ± 5
P(3HP)		37 ± 5	4 ± 4
UHMW-P(3HB)		65 ± 5	78
UHMW-P(3HB)	Stretched	80 ± 5	78
UHMW-P(3HB)	Stretched-annealed	85	78

*^a^* Determined by wide-angle X-ray diffraction.

## 4. Biodegradability of PHA Fibers

PHA fibers have attracted much attention for use as fishing line and surgical sutures. The physical properties of PHA fibers just after molding are stiff and brittle because of secondary crystallization at room temperature. Iwata et al. succeeded in producing strong P(3HB) fibers with a tensile strength of 1.3 GPa and P(3HB-*co*-3HV) fibers with a tensile strength of 1.1 GPa by a new drawing method [11,78]. BOD biodegradability of P(3HB) fibers using Arakawa river water increased with time and reached about 80% within 14 days. Since the weight loss biodegradability of fibers after 28 days was 100%, it was confirmed that strong P(3HB) fibers are completely degraded by microorganisms [79].

**Figure 12:**
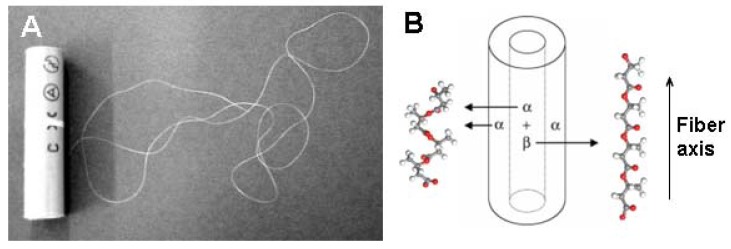
Cold-drawn annealed UHMW-P(3HB) fiber and schematic display of the core-sheath structure of P(3HB) monofilament with two kinds of molecular conformations [11,79,83]. Reproduced with permission from Iwata *et al.*, *Macromol. Rapid Commun.*; published by Wiley-VCH Verlag GmbH & Co. KGaA, 2004.

X-ray patterns for a bundle of ten pieces of P(3HB) and P(3HB-*co*-3HV) fibers showed reflections from both the α-form (2_1_ helix conformation) [80,81] and the β-form (planar zigzag conformation) [82] of P(3HB) simultaneously (Figure 12). It is well known that P(3HB) crystallizes as an orthorhombic crystal system with a space group of P2_1_2_1_2_1_ (α-form), [80,81] and that the β-form is introduced by the orientation of free chains in the amorphous regions between α-form lamellar crystals [82,83,84]. The distribution of the two types of molecular conformations in monofilaments was studied by micro-beam X-ray diffraction experiments, indicating that strong two-step-drawn UHMW-P(3HB) fiber has a coresheath structure with only a 2_1_ helix conformation (α-form) in the sheath region, and with both the planar zigzag conformation (β-form) and 2_1_ helix conformation (α-form) in the core region. On the other hand, the result of one-step-drawn P(3HB-*co*-8mol%-3HV) mono-filament shows that the strong one-step-drawn fibers have no core-sheath structure.

**Figure 13:**
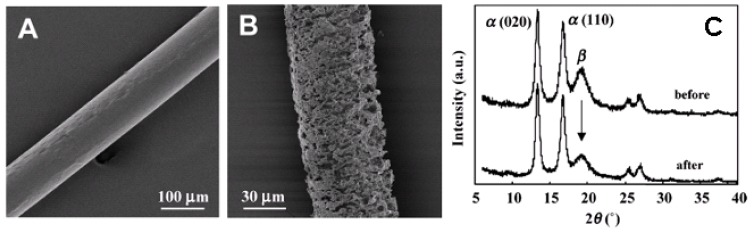
Partial enzymatic degradation of PHA fibers. SEM of drawn annealed P(3HB) fibers before (A) and after partial enzymatic degradation by *R*. *pickettii* T1 (B). (C) Intensity profiles of equatorial lines in X-ray fiber diagrams before and after partial enzymatic degradation by PHA depolymerase purified from *R*. *pickettii* T1 at 37 °C. The α and β indicate the reflections derived from α-form and β-form crystal, respectively [83]. Reproduced with permission from Iwata *et al.*, *Macromolecules*; published by ACS, 2006.

Enzymatic degradation of the cold-drawn and two-step-drawn UHMW-P(3HB) fibers was performed in an aqueous solution containing extracellular PHA depolymerase from *R*. *pickettii* T1 at 37 ºC [83,85]. Figure 13 shows the scanning electron micrographs and WAXD patterns after partial enzymatic degradation for the P(3HB) fibers prepared by stretching after isothermal crystallization. The surface of the P(3HB) fiber before enzymatic degradation was smooth throughout the fiber as shown in Figure 13A. However, the surface of the P(3HB) fiber after partial enzymatic degradation was irregular, and has many fine elliptic voids along the drawing direction (Figure 13B). This finding revealed that the high-strength P(3HB) fiber was degraded by the extracellular PHA depolymerase, and that the enzymatic erosion progressed rapidly and uniformly from the amorphous region at the surface. The morphology of the fibers after partial enzymatic degradation indicates the existence of crystal domains, because the enzymatic degradation progresses initially from the amorphous region at the surface. The X-ray diffraction patterns of the P(3HB) fiber before enzymatic degradation showed the reflections that are attributed to a highly oriented α-form and the presence of β-form. However, the reflection of the β-form disappeared in the X-ray diffraction pattern of the P(3HB) fiber after enzymatic degradation for 1 h. While the intensities of α-form crystals remained unchanged before and after enzymatic degradation, the intensity of β-form decreased, despite β-form existing in core region. These results demonstrated that the enzymatic erosion rate of β-form with the planar zigzag conformation is faster in comparison with α-form with the 2_1_ helix conformation for the P(3HB) fibers [83,85]. Further, the rate of enzymatic erosion can be controlled by the molecular conformation, despite the same chemical structure. 

**Figure 14:**
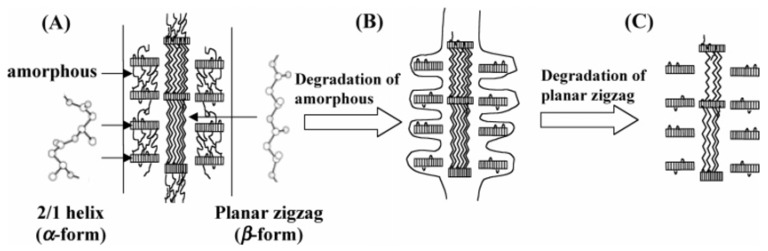
Schematic display of enzymatic degradation behavior of P(3HB) fibers with two kinds of molecular conformations: α-form (2_1_ helix conformation) and β-form (planar zigzag conformation) [83]. Reproduced with permission from Iwata *et al*., *Macromolecules*; published by ACS, 2006.

The degradation mechanism of β-form and α-form in P(3HB) monofilament is presented in Figure 14. Figure 14A shows a schematic of the highly ordered structure of P(3HB) fiber with two kinds of molecular conformations. The sheath region consists of two domains that are lamellar crystals with 2_1_ helix conformation (α-form) with an amorphous region between these lamellar crystals. On the other hand, in the core region, β-form domains exist between lamellar crystals alongside highly oriented amorphous chains. Figure 14B demonstrates that the enzymatic degradation progresses from the amorphous region of the material surface, and then the enzymatic degradation of P(3HB) fibers progresses from the amorphous region between α-form lamellar crystals in the fiber surface (sheath region). Further, the enzyme molecules can penetrate inside the fiber by degrading the amorphous regions. Molecular chains of β-form may be easily attacked by enzyme molecules rather than those of α-form because of less steric hindrance against the ester bond in planar zigzag conformation compared to the helix conformation. The intensity induced from β-form therefore decreased and α-form crystal remained unchanged after partial enzymatic degradation. In a longer enzymatic degradation test, it is confirmed that whole fibers are completely degraded by PHA depolymerase. 

**Figure 15:**
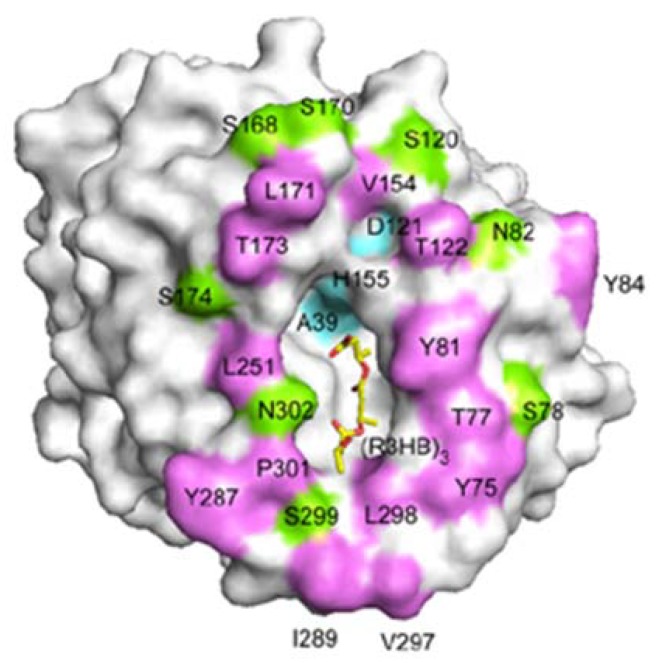
A molecular surface representation of the PHA depolymerase from *P*. *funiculosum* centering on the mouth of the crevice. Positions of solvent-exposed hydrophobic residues (purple), as well as polar (green) and catalytic triad (cyan) residues, are indicated. A model of the 3HB trimer bound in the crevice is shown as a yellow stick model [86]. Reproduced with permission from Hisano *et al.*, *J. Mol. Biol*.; published by Elsevier, 2006.

Figure 15 shows the crystal structures of PHA depolymerase from *Penicillium funiculosum* and its S39A mutant complexed with the methyl ester of a trimer substrate of 3HB revealed using synchrotron X-ray radiation [86]. The catalytic residues Ser39, Asp121, and His155 are located at topologically conserved positions, and then the main chain amide groups of Ser40 and Cys250 form an oxyanion hole. A crevice is formed on the surface of the enzyme, to which a single polymer chain can be bound by predominantly hydrophobic interactions with several hydrophobic residues. Therefore, β-form (planar zigzag) conformation is preferentially bound in the active site crevice in comparison with the α-form. This result supports the faster β-form degradation, which agrees with the experimental results of enzymatic degradation of PHA fibers.

## 5. Perspectives

In this review, the studies on relations between the enzymatic degradability and the solid-state structure of PHA materials were summarized. The information integrated in this review concerning the degradation processes of PHA materials will enable the design and synthesis of biodegradable polymers with controlled degradation rates in the natural environment. For the practical use of PHA as biodegradable materials, their degradation rate should be controlled by chemical structure and solid-state properties. As mentioned in this review, we therefore need to control degradation of PHA lamellar crystals, which is the rate-determining step, in order to regulate overall degradation rates of PHA materials.
